# Overexpression of miR-223 Promotes Tolerogenic Properties of Dendritic Cells Involved in Heart Transplantation Tolerance by Targeting Irak1

**DOI:** 10.3389/fimmu.2021.676337

**Published:** 2021-08-05

**Authors:** Shun Yuan, Yuanyang Chen, Min Zhang, Zhiwei Wang, Zhipeng Hu, Yongle Ruan, Zongli Ren, Feng Shi

**Affiliations:** ^1^Department of Cardiovascular Surgery, Renmin Hospital of Wuhan University, Wuhan, China; ^2^Cardiovascular Surgery Laboratory, Renmin Hospital of Wuhan University, Wuhan, China; ^3^Central Laboratory, Renmin Hospital of Wuhan University, Wuhan, China

**Keywords:** miR-223, dendritic cells, Irak1, heart transplantation, immunosuppression

## Abstract

Dendritic cells (DCs) are key mediators of transplant rejection. Numerous factors have been identified that regulate transplant immunopathology by modulating the function of DCs. Among these, microRNAs (miRNAs), small non-coding RNA molecules, have received much attention. The miRNA miR-223 is very highly expressed and tightly regulated in hematopoietic cells. It plays an important role in modulating the immune response by regulating neutrophils and macrophages, and its dysregulation contributes to multiple types of immune diseases. However, the role of miR-223 in immune rejection is unclear. Here, we observed expression of miR-223 in patients and mice who had undergone heart transplantation and found that it increased in the serum of both, and also in DCs from the spleens of recipient mice, although it was unchanged in splenic T cells. We also found that miR-223 expression decreased in lipopolysaccharide-stimulated DCs. Increasing the level of miR-223 in DCs promoted polarization of DCs toward a tolerogenic phenotype, which indicates that miR-223 can attenuate activation and maturation of DCs. MiR-223 effectively induced regulatory T cells (Tregs) by inhibiting the function of antigen-presenting DCs. In addition, we identified Irak1 as a miR-223 target gene and an essential regulator of DC maturation. In mouse allogeneic heterotopic heart transplantation models, grafts survived longer and suffered less immune cell infiltration in mice with miR-223-overexpressing immature (im)DCs. In the miR-223-overexpressing imDC recipients, T cells from spleen differentiated into Tregs, and the level of IL-10 in heart grafts was markedly higher than that in the control group. In conclusion, miR-223 regulates the function of DCs *via* Irak1, differentiation of T cells into Tregs, and secretion of IL-10, thereby suppressing allogeneic heart graft rejection.

## Introduction

Heart transplantation is currently the most effective treatment for end-stage heart failure. However, immune rejection is a critically important potential complication and the largest risk factor for chronic rejection and dysfunction of a transplanted allograft. Despite the effectiveness of immunosuppressive drugs in improving short-term outcomes of organ transplantation, the long-term use of immunosuppressive drugs inevitably leads to toxic side effects, which makes long-term patient and graft survival suboptimal. Thus, there is an urgent need for an improved approach to treatment that can induce allograft-specific tolerance in the recipient and enable long-term graft survival while avoiding the use of immunosuppression that causes toxic side effects. More importantly, recognition of novel immunosuppressive molecules and signaling pathways will facilitate the development of new immunological tolerance strategies for heart transplantation.

As potent antigen presenting cells (APCs), dendritic cells (DCs) play pivotal roles in initiating antigen-specific immune responses and are key mediators in transplantation immunopathology (1). Following organ transplantation, DCs are essential for the induction of an immune response to the graft or in maintaining immunological tolerance, depending on their activation state and maturation status (2), for which they are classified as either mature (mDCs) or immature (imDCs). Mature DCs, which express high levels of major histocompatibility complex (MHC) and costimulatory molecules (CD40, CD80, CD86) on their surface (3–5), can promote T-cell activation and accelerate graft rejection (6). However, immature DCs with a deficiency in MHC and costimulatory molecules cause immune deviation from Th17 cells to regulatory T cell (Treg) subsets, provoke antigen-specific T-cell hyporesponsiveness, and induce antigen-specific tolerance (2, 7). Immature DCs (imDCs) are induced by numerous immunosuppressive agents, including cytokines (IL-10, TGF-β) and endogenous immunosuppressants (glucocorticoids) (8). They play important roles in inducing peripheral tolerance *via* specific mechanisms, including induction of Tregs, promotion of anti-inflammatory cytokine secretion (9), suppression of effector T cells, and negative modulation of Th1/Th2 immune responses (10). Studies have shown that infusion of imDCs has become a promising modality for immunosuppression in solid organ transplantation (11–13). Therefore, dynamic regulation of DC maturation is crucial for influencing the balance between tolerance and immunity in T-cells.

MicroRNAs (miRNAs) are a group of highly conserved, small single-stranded non-coding RNAs that range from 20 to 25 nucleotides in length. MiRNAs regulate gene expression by base-pairing with complementary sites on target mRNAs, thereby inducing translation blockage and/or mRNA degradation (14–17), and play crucial roles in cellular differentiation, immune modulation, and pathogenic conditions. Numerous studies in multiple model organisms have demonstrated that miRNAs are involved in the pathogenesis of immune diseases, including graft rejection (18–20). Among the known miRNAs, miR-223 is a potent regulator of some inflammatory responses. Studies have shown that miR-223-deficient mice have profound neutrophilia in the peripheral blood and spontaneously develop lung inflammation marked by neutrophil infiltration (21). In addition, abnormal expression of miR-223 is involved in various pathogenic conditions such as type II diabetes, acute lung injury, rheumatoid arthritis, and inflammatory bowel disease (22–25). Furthermore, in experimental autoimmune encephalomyelitis models, miR223^−/−^ mice exhibited a decreased severity of spinal cord lesion and lessening in neurological symptoms accompanied by a significant decrease in spinal cord inflammation and anti-inflammatory APC infiltration. The investigators suggested that miR-223 deficiency protected against spinal cord inflammation by inhibiting bone marrow–derived DC (BMDC) activation (26, 27). Finally, Van Caster and colleagues have reported that miR-223 is overexpressed in the serum of liver transplant patients (28). Thus, the function of miR-223 appears complex. However, the role of miR-223 in the regulation of heart transplantation is still unknown. Considering the complexity of miRNA-223 in the regulation of the immune system, the goal of the current study was to determine whether miR-223 can mediate the function of native DCs, and if so, to explore the underlining mechanisms that regulate the host immune response to allogeneic heart grafts.

In this study, we found that expression of miR-223 increased in the serum of patients and mice following heart transplantation. Similar results were also observed in mouse allografts. Involvement of miR-223 in a heart transplant rejection model was identified, and the contribution of miR-223 to tolerogenic function of DCs *via* inhibition of Irak1 was demonstrated for the first time. Transfusion of miR-223-overexpressing imDCs into mice attenuated rejection of allogeneic heart grafts. Our results suggest that miR-223 might serve as a potential diagnostic biomarker and new therapeutic target for allogeneic heart graft rejection.

## Materials and Methods

### Animals

Male C57BL/6 (H-2b) and BALB/c (H-2d) mice, aged 8–12 weeks, were purchased from the Department of Laboratory Animal Science (Peking University Health Science Center, Beijing, China). All mice were maintained under specific pathogen-free conditions. The Ethical Committee of Renmin Hospital of Wuhan University approved the animal study protocol (WDRM-20171104). The handling of all animals was performed in accordance with the Wuhan Directive for Animal Research and Current Guidelines for the Care and Use of Laboratory Animals published by the National Institutes of Health. All experiments with animals took place in the Animal Experiment Center of Renmin Hospital of Wuhan University.

### Human Studies

Blood from 30 patients who had undergone heart transplant surgery in Renmin Hospital of Wuhan University (Wuhan, Hubei Province, China) was harvested before and after heart transplantation. The blood was immediately centrifuged at 3,000 rpm for 5 min, and the serum was harvested and saved at −80°C for further study. Ours was a case-control study that was approved by the ethics committee of Renmin Hospital of Wuhan University (WDRY2015-K021) and adhered to the tenets of the Declaration of Helsinki. All study subjects received verbal and written information about the study and signed a written consent form prior to participation.

### Cell Isolation and Culture

T cells and DCs were isolated from the spleens of BALB/c mice and purified by magnetic bead-based cell sorting (Miltenyi Biotec). CD4^+^ T cells then were separately cultured in RPMI 1640 medium (HyClone, South Logan, UT, USA) containing 10% fetal bovine serum (Sciencell, Carlsbad, CA, USA) and 1% penicillin/streptomycin. 

### Culture and Transfection of Bone Marrow–Derived DCs

Bone marrow–derived DCs (BMDCs) were generated from male C57BL/6 (H-2b) mice. The cells were grown in RPMI-1640 medium with 20 ng/ml rmGM-CSF (PeproTech) and 10 ng/ml rmIL-4 (PeproTech) at 37°C with 5% CO_2_. On day 6 of cell culture, BMDCs were selected using CD11c magnetic microbeads (Miltenyi Biotec, Bergisch Gladbach, Germany). The purified immature (BMDCs) were then plated at a density of 1 × 10^6^ cells/ml in six-well cell culture plates and transfected with miR-223 mimic, miR-223 inhibitor, miR-223 negative control, or Irak1 short interfering RNA (siRNA) (50 nmol/L; GenePharma, China) using lipofectamine 2000 (Invitrogen, Carlsbad, CA, USA) for 6 h. On day 7, the BMDCs were treated with lipopolysaccharide (LPS; 100 ng/ml; Sigma) for 12 h to induce maturation.

### Phagocytosis Assay

The phagocytic ability of BMDCs was assessed by the uptake of fluorescein isothiocyanate (FITC)-dextran using flow cytometry as described previously (29). BMDCs (1 × 10^6^ cells) were incubated with FITC-dextran (1 mg/ml; Sigma-Aldrich) in 250 µl basal media for 2 h at 4°C or 37°C. Cells were harvested and washed three times in cold FACS buffer before analysis by flow cytometry.

### Mixed Lymphocyte Reaction (MLR) Assay

As stimulators for allogeneic T cells, BMDCs, isolated from C57BL/6 (H-2b) mice, were treated with mitomycin C (10 μg/ml, Sigma-Aldrich, St. Louis, MO, USA). Splenic T cells, harvested from BALB/c (H-2d) mice, were co-cultured with allogeneic BMDCs at a BMDC:T ratio of 1:10 for 3 days in 96-well plates under standard conditions to assess T-cell differentiation. The splenic T cells were stained with carboxyfluorescein succinimidyl ester (CFSE) (10 µM/ml, BestBio, China) and co-cultured with allogeneic BMDCs as previously described to assess T-cell proliferation. In another MLR study, splenocytes (2 × 10^6^), harvested from three groups of recipient mice, were stained with CFSE (10 µM/ml, BestBio, China) and were responders, while splenocytes (6 × 10^6^, 3:1) from C57BL/6 (H-2b) mice treated with mitomycin C (10 μg/ml, Sigma-Aldrich, St. Louis, MO, USA) were stimulators. CFSE was used to assess T-cell proliferation.

### Flow Cytometry

Cells were harvested and stained with FITC-anti-CD80 mAb, FITC-anti-CD86 mAb, and FITC-anti-MCH-II mAb (all from eBioscience, San Diego, CA, USA). A fluorescence-activated cell sorter (FACS) was used for analysis. For Treg cells analyzed by flow cytometry, splenocytes and cells in the MLR supernatant were stained with FITC-anti-CD4 and phycoerythrin(PE)-anti-CD25, followed by APC-anti-Foxp3 staining buffer (all from eBioscience) according to the manufacturer’s instructions. The stained cells were analyzed using the FACSCanto II system (BD Biosciences) to determine the percentage of CD4^+^CD25^+^Foxp3^+^cells among CD4^+^ T cells, and the data were analyzed using FlowJo software.

### Quantitative Reverse Transcriptase Polymerase Chain Reaction

Total RNA was exacted using TRIzol reagent (Qiagen, Valencia, CA, USA) and used in reverse-transcription and PCR amplification reactions. Levels of glyceraldehyde-3-phosphate dehydrogenase (GAPDH) PCR production were assessed to ensure that equal amounts of RNA were input to the RT-PCR reaction. Real-time quantitative RT-PCR was performed using the CFX96 Real-Time PCR System with specific primers and software (Bio-Rad, Hercules, CA, USA). For miRNA studies, we quantified the level of miR-223 in cells and tissues with real-time quantitative RT-PCR, and small nuclear RNA U6 was used as an endogenous control. We also tested the level of miR-223 in the serum of heart transplant patients and recipient mice, and cel-miR-39 was used as an endogenous control. All primers, cel-miR-39, and miRNA mimics and inhibitors were purchased from RiboBio (Guangzhou, China). The primer sequences used for RT-PCR are shown in **Table 1**.

**Table 1:** Primer sequences used for RT-PCR.

Name	Sequences
miR-223	miR-223-3p forward: GCGTGTATTTGACAAGCTGAGTTmiR-223-3p reverse: GTGTCAGTTTGTCAAATACCCCA
U6	U6 forward: GCTTCGGCAGCACATATACTAAAATU6 reverse: CGCTTCACGAATTTGCGTGTCAT
GAPDH	GAPDH forward: CCTTCATTGACCTCAACTACATGGGAPDH reverse: CTCGCTCCTGGAAGATGGTG
Irak1	Irak1 forward: GCCCTTTGGCTCTATTTGGGIrak1 reverse: TCTGAGGCTCATCCAGCAAAG
IL-6	IL-6 forward: CAGAAGGAGTGGCTAAGGACCAIL-6 reverse: ACGCACTAGGTTTGCCGAGTAG
IL-10	IL-10 forward: GACCAGCTGGACAACATACTGCTAAIL-10 reverse: GATAAGGCTTGGCAACCCAAGTAA
IL-12	IL-12 forward: GTCCTCAGAAGCTAACCATCTCCIL-12 reverse: CCAGAGCCTATGACTCCATGTC

### Western Blotting

To analyze protein expression in BMDCs after the various treatments, total protein was extracted from BMDCs using RIPALysis buffer (P0013B, Beyotime Corporation, China) containing a cocktail of protease inhibitors (Roche 4693159001, Merck Corporation, USA) and phenylmethanesulfonyl fluoride (MA0001, Dalian Meilune Biotechnology Corporation, China). Protein concentrations were measured with a BCA assay kit (P0011, Beyotime Corporation, China). Equal quantities of protein samples were denatured and separated by 8–12% SDS-PAGE. Proteins were then transferred to Polyvinylidene Fluoride (PVDF) membranes and incubated in PBS containing 5% skimmed milk to block non-specific binding. Membranes were then incubated overnight at 4°C in a solution containing primary detection antibody. After being washed with PBS, the membranes were incubated with appropriate secondary detection antibodies, and positive binding was visualized using an Odyssey infrared imager (LI-COR, NE, USA) and quantified according to grayscale values of each band using software included with the imager.

### Enzyme-Linked Immunosorbent Assay (ELISA)

Secretion of cytokines from BMDCs was measured with mouse IL-6, IL-10, and IL-12 ELISA kits according to the manufacturer’s instructions (BD Biosciences, CA, USA).

### MiR-223 Target Gene Prediction and Validation

MiR-223 target gene prediction was conducted with TargetScan Mouse 5.1 (www.targetscan.org) and PicTar (pictar.mdc-berlin.de) (30, 31). To validate the predicted miR-223 targets, a luciferase reporter assay was performed. Two luciferase reporter vectors were constructed: (1) pGL3-Irak1-3’UTR-WT containing the 3’UTR of Irak1 with binding sites for miR-223, and (2) pGL3-Irak1-3’UTR-MU with mutations at the predicted miR-223 binding sites. The reporter constructs pGL3-Irak1-3’UTR-WT, pGL3-Irak1-3’UTR-MU, or pRL-TK (Promega, Madison, WI, USA) were co-transfected with miR-223 mimic oligonucleotides into HEK293 cells. Forty-eight hours after co-transfection, the activities of Renilla luciferase were measured with a dual-luciferase reporter system (Promega) and normalized to the internal control firefly luciferase activity. The repressive effects of miR-223 on gene targets were plotted as the percentage of repression of three biological repeats (each biological repeat contained three technical repeats).

### Heart Transplantation

Heterotopic cervical heart transplantation in the mouse was performed as described previously (32). Briefly, heart grafts were harvested from donor mice and transplanted into the cervical region of recipient mice by anastomosing the aorta and pulmonary artery of the graft end-to-end to the recipient’s right carotid artery and external jugular vein, respectively, with a cuff technique. Graft survival was monitored by daily cervical palpation, and graft rejection was defined as cessation of palpable heartbeats, verified by cervical skin dissection. MiR-223-overexpressing imBMDCs, normal imBMDCs, or PBS were injected to the recipients 7 days before surgery at a dose of 1 × 10^7^ plaque-forming units/mouse/day. At 7 days after transplantation, partial recipient mice were sacrificed, and heart allografts and spleens were harvested for further study. The remainder were used to measure survival time of the heart grafts.

### Tissue Histology

The heart grafts were harvested, fixed in formalin, and embedded in paraffin. Tissue blocks were sectioned at 2 μm, and slides were baked at 60°C for 1 h, deparaffinized, and rehydrated, followed by staining with hematoxylin and eosin (H&E) and evaluated by light microscopy (Nikon Eclipse 80i; Nikon, Tokyo, Japan). To quantify levels of immunological rejection, the presence of vasculitis, thrombosis, hemorrhage, and inflammatory cell infiltration was observed in a minimum of three random microscopic fields.

### Statistical Analysis

Student’s t test and 1-way ANOVA were used as indicated. Differences in animal survival (Kaplan-Meier survival curves) were analyzed with the log-rank test. All statistical analyses were performed using GraphPad Prism 6 (GraphPad Software, La Jolla, CA, USA), and p < 0.05 was considered significant.

## Results

### MiR-223 Is Involved in Heart Transplant Rejection and DC Maturation

**Figure 1:**
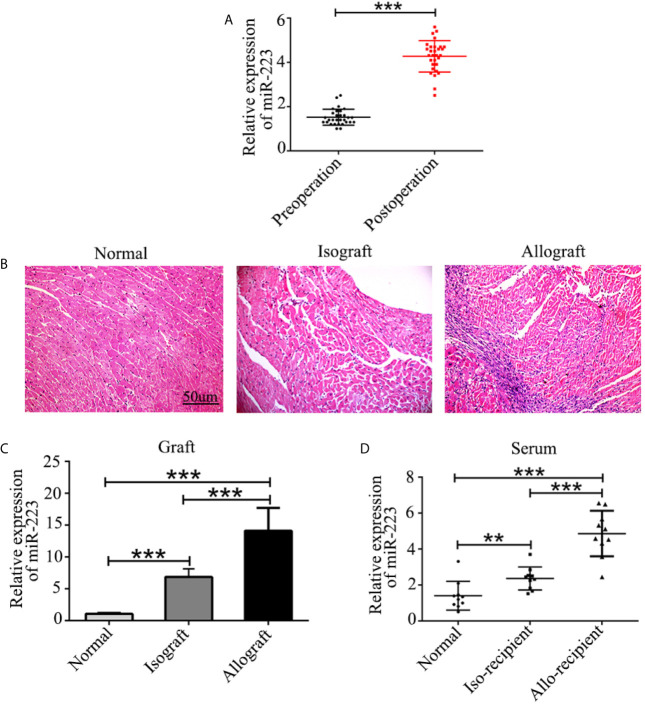
Expression of miR-223 in heart transplant patients and mice. **(A)** Concentration of miR-223 in the serum of patients before and 7 after days heart transplantation. **(B)** Pathological structure of normal heart isografts and allografts indicated by H&E staining. **(C, D)** Expression of miR-223 in heart grafts **(C)** and serum **(D)** of mice 7 days after heart transplantation. Data are presented as mean ± SD and represent six experiments. **P < 0.01, ***P < 0.001.

Serum was harvested from patients both before and 7 days after heart transplantation, and the concentration of miR-223 was measured by quantitative RT-PCR. The results revealed increased expression of miR-223 in the serum of heart transplant patients (**Figure 1A**). Similar results were obtained in allografts and serum from allo-recipient mice (**Figures 1B–D**). These data indicate that miR-223 may be involved in regulating allogeneic heart graft rejection.

**Figure 2:**
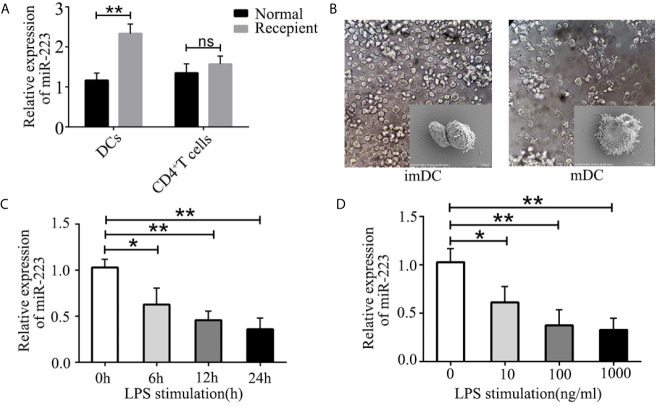
Role of miR-223 in DC maturation. **(A)** Expression of miR-223 in DCs and CD4^+^ T cells from the spleens of normal and recipient mice. **(B)** Typical features of imBMDCs and mBMDCs. **(C, D) **miR-223 expression in BMDCs stimulated with LPS (100 ng/ml) for the indicated times **(C)** or with the indicated concentration of LPS for 12 h **(D)**. Data are presented as mean ± SD and represent three experiments. *P < 0.05; **P < 0.01; ns, not significant.

It has been shown that DCs and CD4^+^ T cells play crucial roles in organ transplant rejection. We hypothesized that miR-223 may be involved in regulating DC and CD4^+^ T cell function during organ transplant rejection. To confirm the role of miR-223 in immune cells, we isolated DCs and CD4^+^ T cells from the spleens of BALB/c mice and assessed the expression of miR-223 in DCs and CD4^+^ T cells. In the heart transplant group, expression of miR-223 was significantly higher in DCs (**Figure 2A**). However, no significant changes in miR-223 expression were observed in CD4^+^ T cells.

To further investigate the mechanism of miR-223 in regulating allogeneic heart graft rejection, we speculated that miR-223 might play a crucial role in regulating the functional states of DCs and thus in mediating heart transplant rejection. To verify our hypothesis, we cultured BMDCs derived from bone marrow cells of male C57BL/6 (H-2b) mice (**Figure 2B**) and measured expression of miR-223 by RT-PCR of BMDCs treated with 100 ng/ml LPS for 0, 6, 12, and 24 h. We found that miR-223 decreased in a time-dependent manner in cells treated with 100 ng/ml LPS (**Figure 2C**). We also compared the effects of treatment with 10, 100, and 1,000 ng/ml LPS for 12 h and found that miR-223 was maximally downregulated by 100 ng/ml LPS (**Figure 2D**). Taken together, these results indicated that miR-223 was involved in DC maturation.

### MiR-223 Promotes DCs Polarization Towards a Tolerogenic DCs

**Figure 3:**
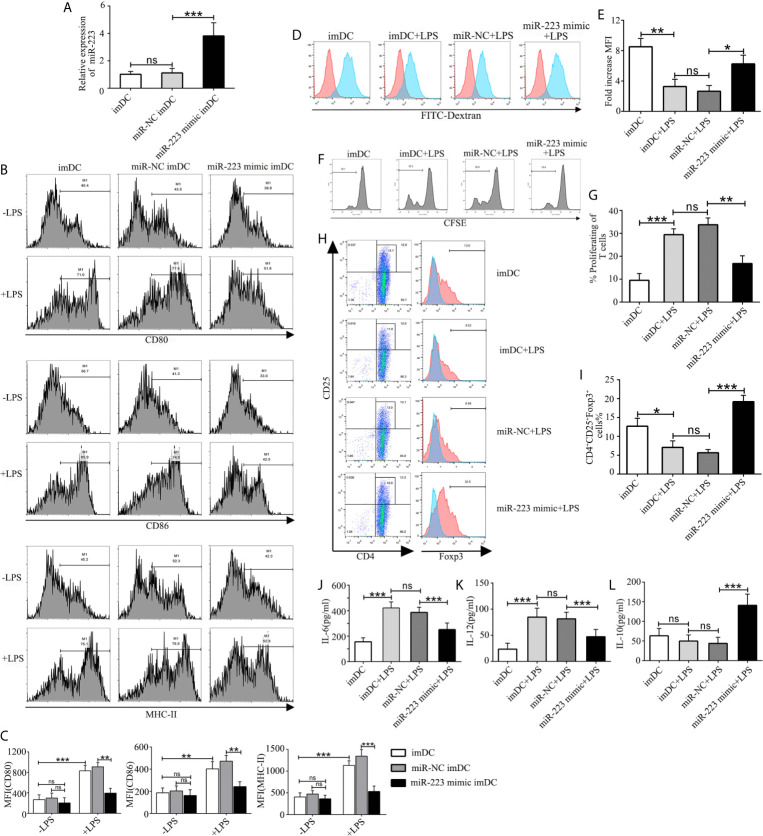
Overexpression of miR-223 promotes tolerogenic properties of DCs. **(A)** Expression of miR-223 in cells transfected with the miR-223 mimic or the miR-223 negative control for 48 h (real-time quantitative RT-PCR). **(B)** imBMDCs, miR-NC-imBMDCs, and miR-223 mimic-imBMDCs were cultured for 12 h in the presence or absence of LPS (100 ng/ml) and then stained with directly conjugated MHC-II, CD86, or CD80 Abs for FACS analysis. **(C)** Mean fluorescence intensity. **(D)** Phagocytic ability of imBMDCs, mBMDCs or miR-NC-imBMDCs, and miR-223 mimic-imBMDCs after LPS stimulation. Phagocytosis of FITC-dextran was measured using FACS. **(E)** Bar chart indicating the fold-increase in mean fluorescence intensity in the four groups. **(F)** Representative FACS results for three independent experiments examining CD4^+^ T-cell proliferation. **(G)** Quantitative assessment of CD4^+^ T-cell proliferation in the four groups. **(H)** Representative FACS results of three independent experiments for the percentage of Tregs. **(I)** Quantitative assessment of the percentage of Tregs in the four groups of Tregs. **(J–L)** Production of cytokines (IL-12, IL-6, IL-10) was analyzed by ELISA in supernatants from BMDC cultures in which imBMDCs, miR-NC-imBMDCs, and miR-223 mimic-imBMDCs were cultured for 12 h in the absence or presence of LPS (100 ng/ml). Data are presented as mean ± SD and represent three experiments. *P < 0.05; **P < 0.01; ***P < 0.001; ns, not significant.

In order to explore the role of miR-223 in regulation of DC function, imBMDCs were transfected with a miR-223 negative control or a miR-223 mimic for 6 h before stimulation with LPS to overexpress miR-223 (**Figure 3A**). We found that expression of miR-223 was effectively upregulated by the miR-223 mimic, whereas the miR-NC had no effect. Flow cytometry was then used to test for the presence of the costimulatory markers CD80, CD86, and MHC-II on the surface of the BMDCs. The results showed that expression of CD80, CD86, and MHC-II was unchanged in the three groups (**Figures 3B, C**), which indicated that miR-NC had no impact on the BMDC phenotype.

To further investigate whether overexpression of miR-223 could maintain imDC in an immature state in the presence of exogenous maturation stimuli, imBMDCs, miR-NC-imBMDCs, and miR-223 mimic-imBMDCs were stimulated with LPS (100 ng/ml) for 12 h. We found that imBMDCs and miR-NC imBMDCs expressed high levels of MHC-II, CD86, and CD80 upon LPS stimulation, whereas the miR-223 mimic-imBMDCs retained low levels of these maturation markers (**Figures 3B, C**). Antigen phagocytosis is the first step in antigen presentation, and studies have shown that immature DCs are more aggressively phagocytotic than are mature DCs, which can be assessed by uptake of FITC-dextran. We observed that imBMDCs were more phagocytic than mBMDCs, and miR-223 mimic-imBMDCs more than miR-NC-imBMDCs, as were imBMDCs after LPS stimulation (**Figures 3D, E**). These results suggested that overexpressing miR-223 in imDCs could maintain DCs in an immature state.

It has been reported that the mature state of DCs has a powerful effect on the ability of DCs to stimulate T-cell proliferation and differentiation. Thus, an MLR assay was conducted to determine the effect of DCs on allogeneic T cell proliferation and differentiation. CD4^+^ T cells purified from BALB/c mice were mixed with allogeneic imBMDCs, miR-NC-imBMDCs, and miR-223 mimic-imBMDCs that had been stimulated by LPS. T-cell proliferation decreased (**Figures 3F, G**) and the percentage of Tregs increased (**Figures 3H, I**) when they were co-cultured with LPS-stimulated miR-223 mimic-imBMDCs, but less so with LPS-stimulated imBMDCs and miR-NC-imBMDCs. These results indicate that miR-223 promotes DC polarization toward tolerogenic DCs, which can induce Treg and allogeneic T-cell hyporesponsiveness.

Cytokines play crucial roles in interactions among immune cells, and we therefore examined cytokine secretion in BMDCs. Levels of the anti-inflammatory cytokine IL-10 increased in miR-223 mimic-imBMDCs, but secretion of maturation-associated cytokines such as IL-6 and IL-12 was lower than in LPS-stimulated imBMDCs and miR-NC imBMDCs (**Figures 3J–L**). Together these results suggested that overexpression of miR-223 inhibited LPS-mediated maturation of imDCs but facilitated increased IL-10 secretion.

### MiR-223 Regulates the Expression of Irak1

**Figure 4:**
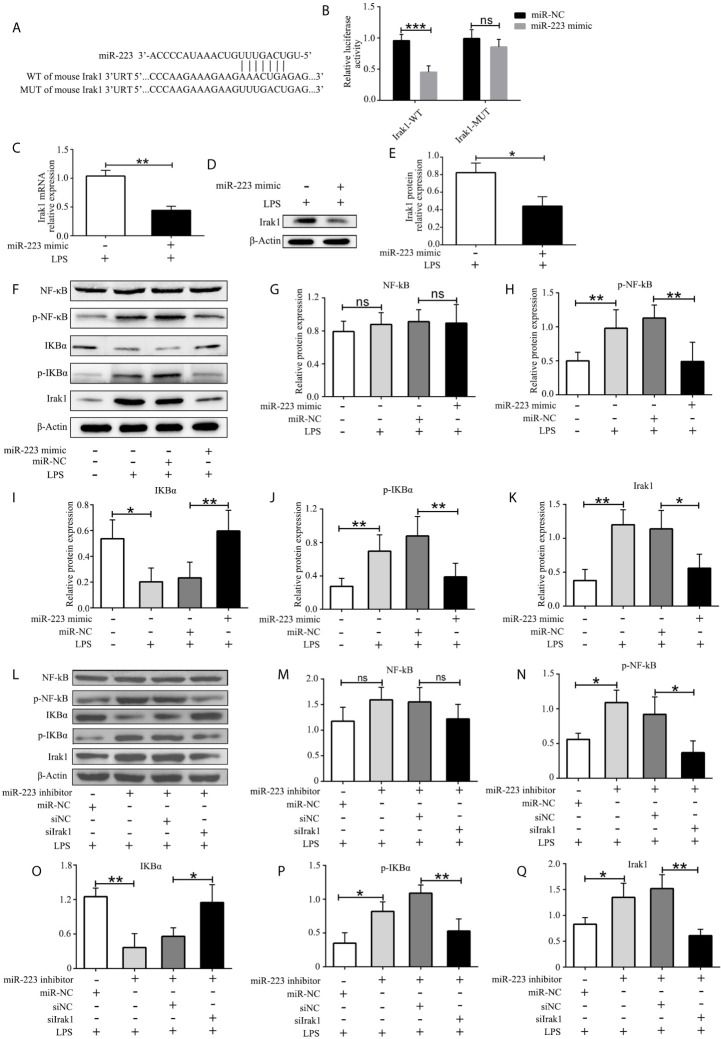
Irak1 is a miR-223 target. **(A)** Predicted miR-223 binding sites in the Irak1 3’-UTR. **(B)** Dual-luciferase reporter gene assay. **(C, D)** Irak1expression was tested by RT-PCR and western blotting. **(E)** Densitometric analysis of Irak1 in murine BMDCs transfected with a miR-223 mimic or control. **(F)** Immunoblot analysis of the indicated total and phosphorylated proteins in lysates of imBMDCs transfected with the miR-223 mimic or miR-NC, and then stimulated with LPS (100 ng/ml) for 12 h. **(G–K)** Densitometric analysis of NF-kB, p-NF-kB, IKBα, p- IKBα, and Irak1 in lysates of imBMDCs transfected with the miR-223 mimic or miR-NC, and then stimulated with LPS (100 ng/ml) for 12 h. **(L)** Immunoblot analysis of the indicated total and phosphorylated proteins in lysates of imBMDCs transfected with miR-223 inhibitor, miR-NC, siNC, and siIrak1, and then stimulated with LPS (100 ng/ml) for 12 h. **(M–Q)** Densitometric analysis of NF-kB, p-NF-kB, IKBα, p- IKBα, Irak1 in lysates of imBMDCs transfected with miR-223 inhibitor, miR-NC, siNC, and siIrak1, and then stimulated with LPS (100 ng/ml) for 12 h. Data are presented as mean ± SD and represent three experiments. *P < 0.05; **P < 0.01; ***P < 0.001; ns, not significant; UTR, untranslated region; WT, wild type; MUT, mutant.

To better understand the mechanism of miR-223 in regulating the function of DCs, we used multiple target gene prediction algorithms, including TargetScan Mouse 5.1 and PicTar, to screen for miR-223 target genes. Irak1 was identified as a genuine target of miR-223 (**Figure 4A**). To further confirm this conclusion, luciferase assays were conducted in HEK293 cells. Luciferase activity was repressed in cells transfected with constructs containing 3^’^-untranslated regions with miR-223 binding sites in the presence of miR-223, whereas these inhibitory effects were not observed using constructs with miR-223 binding site mutations (**Figure 4B**). What’s more, expression of Irak1 was lower in BMDCs that were transfected with the miR-223 mimic and stimulated with LPS (100 ng/ml for 12 h) compared with those infected with the miR-223 negative control (**Figures 4C–E**). It has been reported that Irak1 has an important role in regulating the NF-κB signaling pathway, so we next tested whether miR-223 overexpression affected NF-κB signaling in BMDCs. We found that transfection with the miR-223 mimic not only decreased Irak1 expression but also reduced the levels of phosphorylated IκBα and p65 protein upon LPS stimulation, indicative of inhibition of NF-κB pathway activation (**Figures 4F–K**). Taken together, these data indicated that miR-223 might play an important role in mediating DC maturation by inhibiting the NF-κB signaling pathway by targeting Irak1. To further determine whether Irak1 plays a role in miR-223 regulation of the NF-κB signaling pathway, we co-transfected imBMDCs with an miR-223 inhibitor and/or a small interfering RNA targeting Irak1 (siIrak1), and then we examined their influence on LPS stimulation of imBMDCs. We found that the miR-223 inhibitor increased expression of Irak1 and phosphorylated IκBα and p65 protein, whereas co-transfection of the siIrak1 reversed the miR-223 inhibitor-induced increases (**Figures 4L–Q**). These results indicate that miR-223 regulates the NF-κB signaling pathway by targeting Irak1.

### MiR-223 Mediates the Function of DCs by Suppressing Irak1

**Figure 5:**
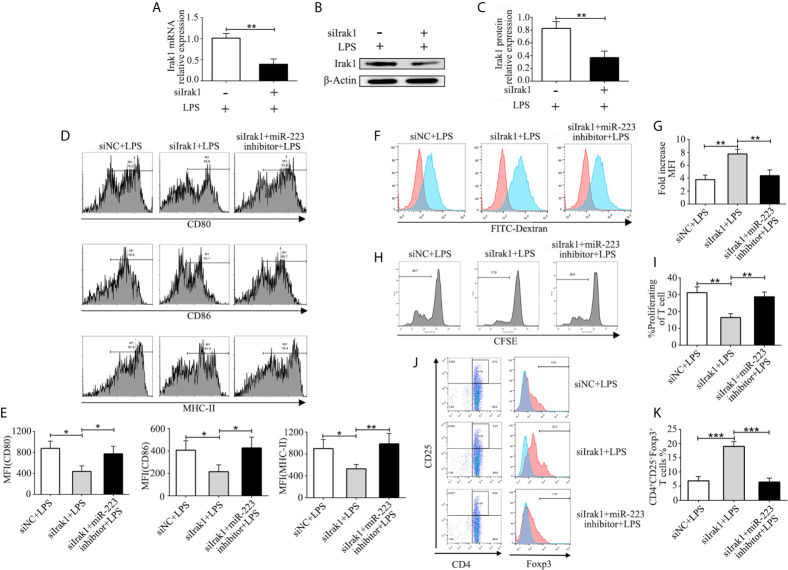
MiR-223 regulates DC function *via* Irak1. BMDCs were transfected with siNC, siIrak1, and siIrak1+miR-223 inhibitor for 6 h before being stimulated with LPS. **(A)** The mRNA levels of Irak1 were examined by qRT-PCR. **(B, C)** Protein expression of Irak1 was determined by western blotting. β-actin was used as a loading control. **(D, E)** BMDCs were stained with directly conjugated CD80, CD86, and MHC-II and analyzed by flow cytometry. **(F)** Phagocytic ability of siR-NC imBMDCs, siIrak1-imBMDCs, and siIrak1+miR-223 inhibitor imBMDCs after LPS stimulation as tested by measuring phagocytosis using FACS. **(G)** Quantitative assessment of the phagocytic ability of siR-NC imBMDCs, siIrak1-imBMDCs, and siIrak1+miR-223 inhibitor imBMDCs after LPS stimulation. **(H)** Representative FACS results of three independent experiments for CD4^+^ T-cell proliferation. **(I)** Quantitative assessment of CD4^+^ T-cell proliferation in three groups. **(J)** Representative FACS results of three independent experiments for the percentage of Tregs. **(K)** Quantitative assessment of the percentage of Tregs in three groups. Data are presented as mean ± SD and represent three experiments. *P < 0.05; **P < 0.01; ***P < 0.001.

In order to further investigate the crucial role of Irak1 in regulating DC maturation, we used a small interfering RNA targeting Irak1 (siIrak1) to knock down Irak1 in imBMDCs. Expression of Irak1 in BMDCs infected with siIrak1was significantly reduced compared with siRNA-negative control (siNC) BMDCs (**Figure 5A**). Similar results were obtained by western blotting (**Figures 5B, C**). Meanwhile, in LPS-stimulated BMDCs, Irak1 knockdown significantly decreased levels of the surface markers CD80, CD86, and MHC-II (**Figures 5D, E**), elevated phagocytosis of FITC-Dextran (**Figures 5F, G**), reduced their ability to induce allogeneic T cell proliferation (**Figures 5H, I**), and enhanced Treg differentiation (**Figures 5J, K**). These effects were similar to those of miR-223 mimic transfection. The BMDCs were then transfected with siIrak1 and a miR-223 inhibitor. As expected, the inhibitory effect of Irak1 knockdown on phenotypic and functional maturation of BMDCs was attenuated by the miR-223 inhibitor (**Figures 5D–K**). These results further support the hypothesis that miR-223 plays a critical role in regulation of DC function by Irak1.

### *In Vivo* Transfer of miR-223 Overexpressing imDCs Attenuates Heart Graft Rejection

**Figure 6:**
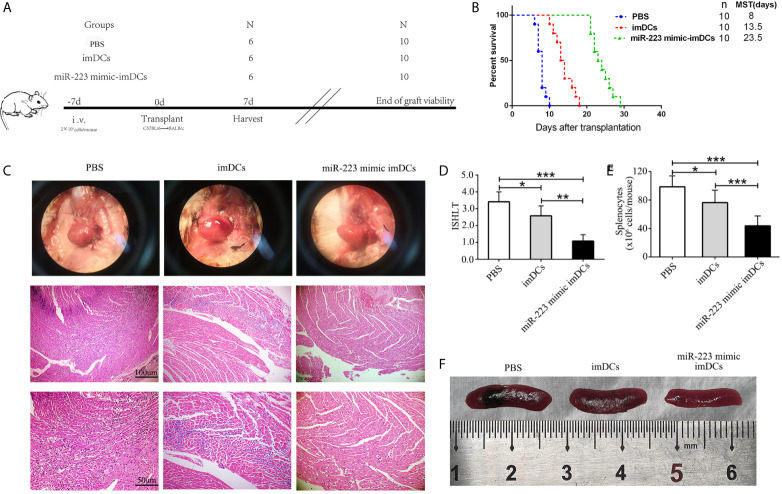
*In vivo* transfer of miR-223-overexpressing imDCs prolongs heart allograft survival. C57BL/6 and BALB/c mice were chosen as donors and recipients, respectively. MiR-223 mimic-transfected imBMDCs, imBMDCs, and PBS were injected. **(A)** Schematic of the study design. **(B)** A Kaplan–Meier survival curve of heart allografts in three groups is presented by day post-transplantation and relative survival (Kaplan–Meier survival analysis). **(C)** Recipient mice in three groups were euthanized at 7 days post-transplantation, and then allograft hearts were harvested and stained with H&E (original magnifications, ×100, ×200). **(D)** Assessment of graft-infiltrating inflammatory cells of heart allografts in three groups at 7 days after transplantation. **(E)** Cell counting of spleens in three groups. **(F)** Morphological characteristics of spleen obtained from three groups. Data are presented as mean ± SD and represent six experiments. *P < 0.05; **P < 0.01; ***P < 0.001.

To determine the influence of miR-223 mimic DCs on allogeneic heart graft rejection, we cultured donor-derived BMDCs and established a cervical heterotopic heart transplantation model. We injected miR-223 mimic-imBMDCs, imBMDCs (2 × 10^6^, 200 µl), or PBS (200 µl) into recipient mice 7 days before heart transplantation (**Figure 6A**). Compared with the PBS group, survival of allografts in recipients pretreated with imBMDCs was modestly prolonged, with median graft survival time (MST) increasing from 8 to 13.5 days. In contrast, miR-223 mimic-imBMDCs exerted a marked tolerogenic effect and prolonged MST to 23.5 days (**Figure 6B**). Partial recipient mice were euthanized, and grafts and spleens harvested. The phenotype and recovery status of heart grafts from the miR-223 mimic-imBMDCs group were much better, and the grafts beat more regularly and strongly than those of the PBS group (**Figure 6C**). Splenomegaly was apparent in the PBS group, while spleens obtained from miR-223 mimic-imBMDCs group mice were the smallest (**Figures 6E, F**). To further confirm that miR-223-overexpressing imBMDCs attenuate heart graft rejection, heart grafts were stained with H&E. Disruption of heart structure, necrosis of partial cardiomyocytes, and infiltration of inflammatory cells were observed in heart grafts from PBS group mice, whereas heart allografts of miR-223 mimic imBMDC group mice showed normal histological features (**Figure 6C**). Besides, we assessed ISHLT scores of the heart allografts according to the standardization of nomenclature in the diagnosis of heart rejection formulated in 1990 (33), and similar results were obtained in the histological grades (**Figure 6D**). In conclusion, miR-223-overexpressing imBMDCs attenuate heart graft rejection, thus prolonging heart graft survival.

### MiR-223-Overexpressing imDCs Induce Tregs and the Anti-Inflammatory Cytokine IL-10 *In Vivo*

**Figure 7:**
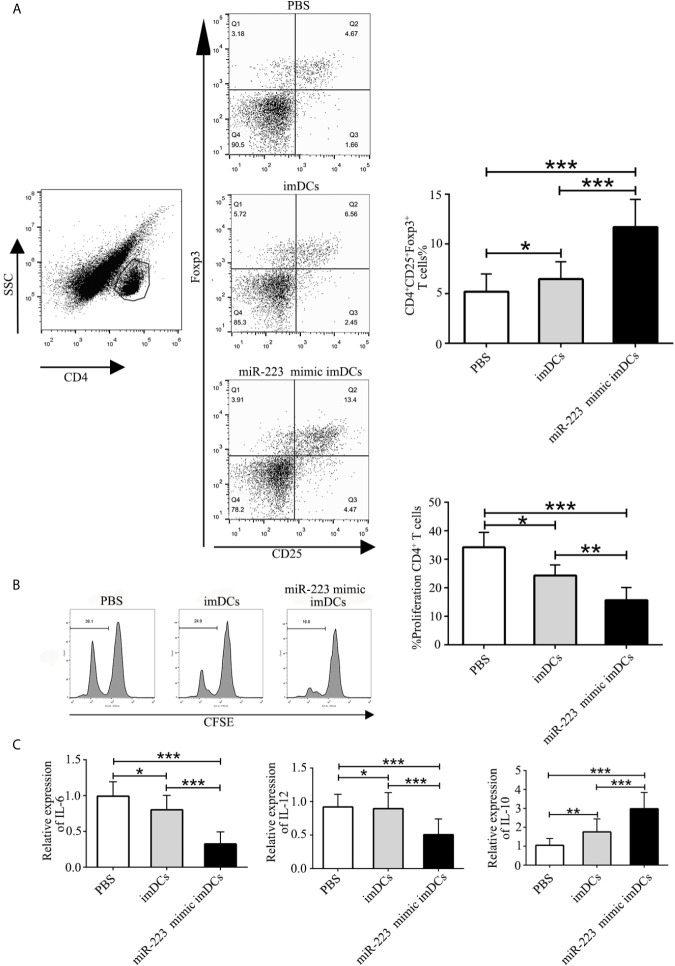
MiR-223-overexpressing DC induces Tregs and the anti-inflammatory cytokine IL-10 in *vivo*. To elucidate the mechanisms underlying miR-223-overexpressing BMDC modulation of heart allograft rejection in *vivo*, splenocytes were isolated from recipient mice 7 days after transplantation and stained with fluorescein-labeled antibodies (CD4, CD25, Foxp3), and subsequently analyzed by flow cytometry. **(A)** An increased proportion of Tregs was observed in miR-223-overexpressing BMDCs. Splenocytes (2 × 10^6^) harvested from recipient mice of three groups and then stained with CFSE were responders, and splenocytes (6 × 10^6^, 3:1) from C57BL/6 mice treated with mitomycin C were stimulators. **(B)** In co-cultured T cells, T-cell proliferation was assessed by flow cytometry. **(C)** Levels of IL-6, IL-12, and IL-10 in heart grafts determined by RT-PCR. Data are presented as mean ± SD and represent six experiments. *P < 0.05; **P < 0.01; ***P < 0.005.

To elucidate the mechanisms by which miR-223 mimic-imBMDCs modulate heart allograft rejection *in vivo*, splenocytes were isolated from recipient mice 7 days after transplantation, stained with fluorescein-labeled antibodies to CD4, CD25, and Foxp3, and subsequently analyzed by flow cytometry. A higher proportion of Tregs was observed in the miR-223 mimic imBMDC group compared with the imBMDC and PBS groups (**Figure 7A**). In the co-cultured T cells, proliferation was assessed by flow cytometry. T-cell proliferation in the miR-223 mimic-imBMDC group was lower than in the imBMDC and PBS groups (**Figure 7B**). In addition, expression of IL-10 in heart grafts from mice transfused with miR-223 mimic-transfected imBMDCs significantly increased compared with that of mice transfused with imBMDCs or PBS, whereas the concentrations of IL-6 and IL-12 were lower in the heart grafts from mice transfused with miR-223 mimic-transfected imBMDCs than in those of mice transfused with imBMDCs or PBS (**Figure 7C**). Our results suggest that miR-223 mimic-imBMDCs attenuate heart allograft rejection by inducing Tregs and the anti-inflammatory cytokine IL-10 *in vivo*.

## Discussion

**Figure 8:**
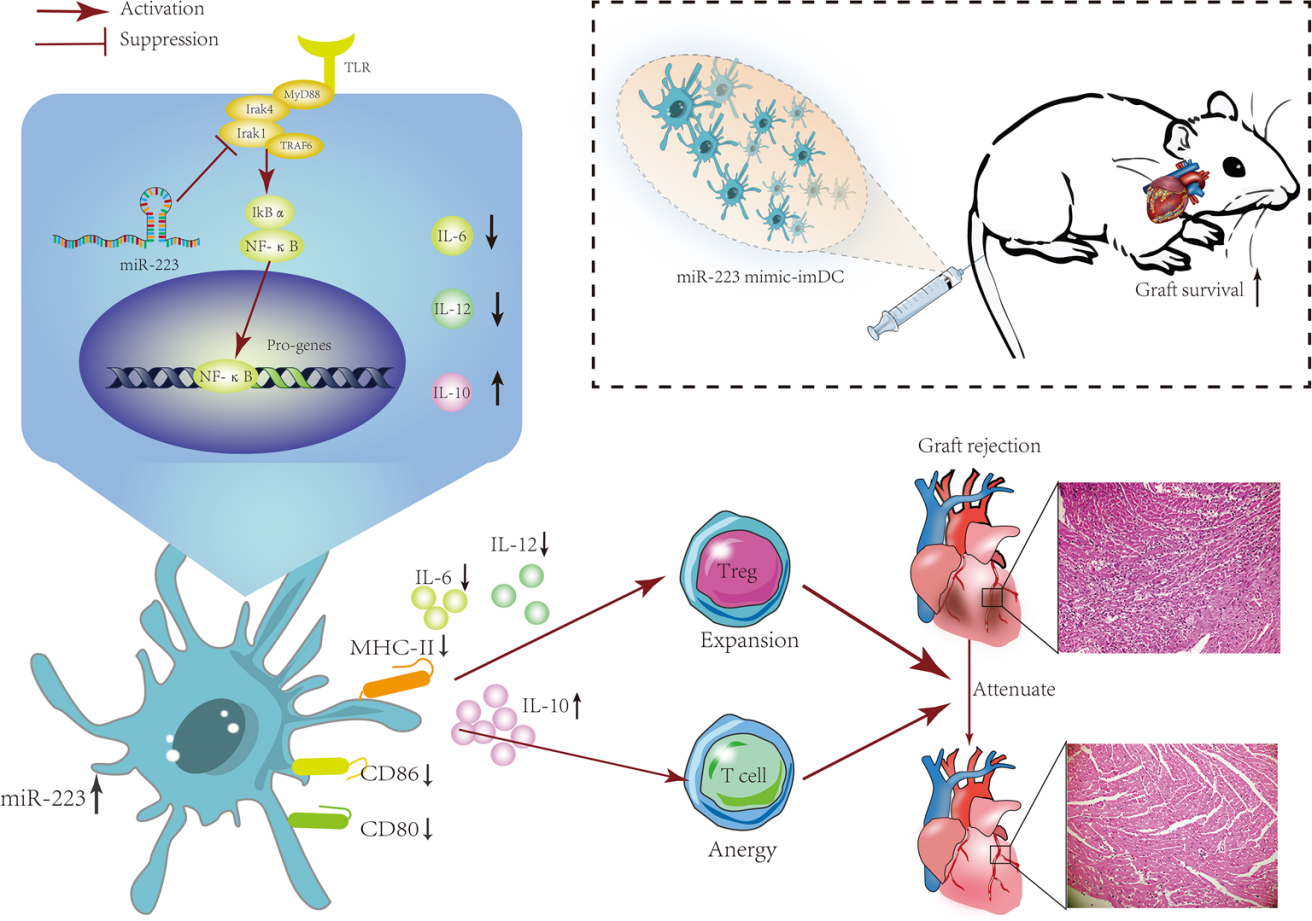
The mechanism of miR-223-overexpressing DCs in modulating heart allograft rejection in *vivo*. Increasing miR-223 expression in BMDCs promotes the polarization of DCs toward a tolerogenic state by targeting Irak1, which promotes graft survival and transplant tolerance in a mouse heart transplantation model by inducing Tregs and increasing IL-10 production.

Here we report increased levels of miR-223 in the serum of heart transplant patients. *In vitro* experiments demonstrated that miR-223 could maintain the immature status of DCs *via* Irak1. *In vivo* experiments showed that imDCs transfected with a miR-223 mimic were associated with a reduced rejection response in recipients. Furthermore, miR-223 induced tolerogenic DCs and increased IL-10 production by targeting Irak1. These findings showed that DCs treated with miR-223 have an immunotolerant effect in a heart transplantation model and can also induce generation of Tregs (**Figure 8**).

MicroRNAs are small highly conserved non-coding RNAs that play crucial roles in multiple physiological and pathological processes by regulating expression of target genes. Emerging evidence has confirmed a prominent role for miRNAs in graft rejection (20, 34, 35). MicroRNA-144 (36), microRNA-10b (37), and miR-142-3p (38) were found to be involved in the process of graft rejection. MiR-223, as a regulator of the immune system, has recently been shown to be involved in several inflammatory diseases, including inflammatory bowel disease and type 2 diabetes (39). However, its role in graft rejection is still unclear. In the present study, we observed a significant increase in miR-223 levels in the serum of heart transplant patients and mice. A similar result was obtained for heart grafts from recipient mice, which indicated that miR-223 plays a role in heart transplant rejection.

DCs play critically important roles in initiating and mediating immune responses, especially in transplant rejection (40, 41). DCs can recognize and capture foreign antigens in grafts, migrate to immune organs, and then induce antigen (Ag)-specific T-cell activation that can damage the graft (42). Therefore, we isolated T cells and DCs from graft recipients and tested for expression of miR-223. The results showed that expression of miR-223 in DCs was higher than in normal controls. LPS blocked miR-223 expression in BMDCs in a dose- and time-dependent fashion. However, increased expression of miR-223 was observed in the serum of heart transplant patients and heart grafts from mice, which might indicate a feedback mechanism. MiR-223 in particular is very highly expressed and tightly regulated in hematopoietic cells (43). The increased expression of miR-223 might involve intercellular communication *via* extracellular vesicles (EVs) released by bone marrow–derived mesenchymal stem cells (BMSCs) and/or other bone marrow–derived blood cells, mainly blood platelets and leukocytes (25, 44, 45). EVs are membrane-bound, nanometer-sized vesicles that are released by cells under normal, stressed, or transformed conditions and are subsequently taken up by a recipient cell (46). EVs containing miR-223 might be absorbed by DCs and act to regulate their maturation, although potential mechanisms remain to be elucidated. Following transfection with miR-223 mimics, we performed a detailed study to investigate the involvement of miR-223 in DC maturation. Overexpression of miR-223 in DCs was observed to inhibit maturation of DCs, induce generation of Tregs, and increase secretion of IL-10 by reducing activation of the NF-kB signaling pathway *via* inhibition of Irak1. Tregs are key regulators of graft rejection and are important in immune tolerance and homeostasis (47). Tregs suppress the Th17/Th1 response during graft rejection and are critically involved in the development of immune tolerance. IL-10 is a powerful immunosuppressive and anti-inflammatory molecule that plays an essential role in inhibiting T-cell activity and limiting immune responses (48). In particular, IL-10 can reduce the inappropriate amplification of Th17 cells and induce Treg differentiation, thus mediating graft immune tolerance. These findings may suggest a new mechanism by which miR-223 controls generation of tolerogenic DCs. To our surprise, our results seem to be in contrast with two previous studies of miR-223 knockout mice suffering from experimental autoimmune encephalomyelitis, which exhibited less CNS autoimmune inflammation and pro-inflammatory APC infiltration than wild-type mice (26, 27). Based on the evidence reported to date, it is clear that miR-223 has an impact on a variety of cellular processes, ranging from cell cycle regulation and invasiveness to hematopoietic differentiation and immune cell function (39). Changes in miR-223 expression in different hematopoietic cell types, as well as genetic interference using knockout and miRNA sponge methods, convincingly support its central role in differentiation, particularly in the myeloid lineage. Thus, miR-223 deficiency may inhibit the differentiation of bone marrow–derived cells and thus prevent activation of APCs, which was supported by a subsequent study (49). The underlying mechanisms of miR-223 in DC activation and differentiation warrant further investigation.

Interleukin-1 (IL-1) receptor-associated kinases (IRAKs), as crucial components in the toll-like receptor (TLR)/IL-1 receptor (IL-1R) signaling pathway, play important roles in mediating immune responses (50). Through kinase and adaptor functions, IRAKs can initiate a cascade of inflammation that ultimately leads to the expression of immune-related genes. Among the IRAK family members, Irak1, which was the first discovered IL-1 receptor kinase, plays a key role in the IL-1 signaling pathway and immune responses. One study has reported that the expression of TNF-α was downregulated in Irak1-deficient macrophages when TLR2 or TLR4 were activated, thereby weakening the response to the lethal effects of sepsis caused by LPS or Gram-negative bacteria (51). Similarly, another study demonstrated that expression of TNF-α and IL-12 in Irak1-deficient spleen lymphocytes was significantly downregulated when TLR9 was activated (52). Therefore, Irak1 plays a major role in inflammatory responses induced by activation of TLR signaling pathways. Furthermore, some studies have reported that LPS mainly promotes the maturation of DCs through TLR signaling pathways (53–55). After binding to TLR4, LPS may regulate the transcription of various genes through the NF-κB pathway, thereby promoting DC maturation. Considering the key role played by Irak1 in the TLR/IL-1R signaling pathway, Irak1 may be related to the maturation of DCs. Recently, regulation of Irak1 by miR-223 has been reported in some diseases. In macrophages infected with *H*. *pylori*, miR-223 has been shown to downregulate Irak1, resulting in inhibition of inflammation (56). In nucleus pulposus cells, miR-223 overexpression could inhibit NF-kB signaling by targeting Irak1 and ultimately suppressing LPS-induced inflammation (57). In the present study, we used multiple target-gene prediction algorithms, including TargetScan Mouse 5.1 and PicTar, to screen for miR-223 target genes, followed by confirmation with luciferase reporter assays, confirming that Irak1 is a target of miR-223 in DCs, in which the miR-223 mimic markedly downregulated Irak1 protein. In addition, we showed that miR-223 overexpression promoted the degradation of Irak1and impaired NF-kB signaling. Moreover, we found that Irak1-silenced BMDCs exhibited similar behaviors as those transfected with the miR-223 mimic, including suppressed expression of MHC-II, CD80, and CD86, and increased IL-10 production, as well as suppression of IL-12 and IL-6 secretion. These data suggest that the miR-223-Irak1 axis regulates DC function and may thus be a potential therapeutic target for reducing heart transplant rejection.

The mechanisms underlying graft rejection are not fully understood. The myocardium is damaged through coordinated interactions between cardiac cells and immune cells. It is accepted that controlling activation of immune cells effectively inhibits progression of graft rejection. Treatment with tolerogenic DCs can improve immune tolerance following organ transplantation by inducing Tregs, increasing anti-inflammatory cytokine secretion, and suppressing T cell activation (58), and hence is regarded as an attractive therapeutic method. In our study, the use of miR-223 mimic-transfected imDCs as a therapeutic tool was examined in a heart transplantation mouse model. Transfusion of miR-223-overexpressing imDCs induced immune tolerance. The number of splenic Tregs increased in recipients. Immunosuppressive response, which correlates with increased proliferation of Tregs, can be induced as a negative regulatory mechanism to protect against a drastic rejection response in the heart transplantation mouse model. Transfusion of miR-223-overexpressing imDCs into the heart transplantation mouse model significantly alleviated inflammatory infiltration and prolonged graft survival in cardiac tissue, thus attenuating graft rejection. These results suggest that miR-223 is a novel regulator of immune tolerance for therapeutic interventions in graft rejection.

## Conclusions

The present study provides new evidence that suggests a critical role for miR-223 in regulating the function of DCs, which directly contributes to the protective effect of miR-223 against graft rejection. Mechanistically, identification of miR-223 and the crucial target gene Irak1 in modulating DC polarization provides novel insights into the network governing organ transplant rejection. We showed that inhibition of Irak1 expression in DCs by miR-223 was linked to decreased expression of MHC II, CD80, and CD86, and increased IL-10 secretion, as well as to suppression of IL-12 and IL-6 production, thereby potentially inducing hyporesponsiveness of T cells, expansion of Tregs, and prolonged allograft survival in a mouse heart transplantation model. These unique observations indicate that miR-223 mimics may serve as a novel approach to prevent and/or treat graft rejection. In the meantime, whether miR-223 can be used in the timely diagnosis and prognosis of graft rejection remains to be further studied.

## Data Availability Statement

The datasets presented in this study can be found in online repositories. The names of the repository/repositories and accession number(s) can be found in the article/Supplementary Material.

## Ethics Statement

The study was approved by the ethics committee of Renmin Hospital of Wuhan University (WDRY2015-K021) and adhered to the tenets of the Declaration of Helsinki. The patients/participants provided their written informed consent to participate in this study. The Ethical Committee of Renmin Hospital of Wuhan University approved the animal study protocol (WDRM-20171104).

## Author Contributions

Design and conduct of the study: SY and MZ. Data collection and analysis: MZ and ZR. Data interpretation: ZH, YR, and FS. Manuscript writing: SY and YR. Manuscript modification and supplementary experiments: SY and CY. All authors contributed to the article and approved the submitted version.

## Funding

This work was supported by the National Natural Science Foundation of China (Grant No. 81570428), Key Support Project of Health Commission of Hubei Province (Grant No. WJ2019Z012), and Guiding Fund of Renmin Hospital of Wuhan University (Grant No. RMYD2018Z07).

## Conflict of Interest

The authors declare that the research was conducted in the absence of any commercial or financial relationships that could be construed as a potential conflict of interest.

## Publisher’s Note

All claims expressed in this article are solely those of the authors and do not necessarily represent those of their affiliated organizations, or those of the publisher, the editors and the reviewers. Any product that may be evaluated in this article, or claim that may be made by its manufacturer, is not guaranteed or endorsed by the publisher.
